# Retrospective analysis of immediate and long-term results of NOSES technique and conventional laparoscopic-assisted resection in patients with colorectal cancer

**DOI:** 10.3389/fsurg.2024.1444942

**Published:** 2024-09-19

**Authors:** Sergei Malev, Hao Zhang, Ziming Yuan, Qingchao Tang, Guiyu Wang, Giorgi Oganezov, Rui Huang, Xishan Wang

**Affiliations:** ^1^ Department of Colorectal Surgery, The Second Affiliated Hospital of Harbin Medical University, Harbin, China; ^2^ Department of Breast Surgery, The Affiliated Tumor Hospital of Harbin Medical University, Harbin, China; ^3^ Department of Colorectal Surgery, National Cancer Center/National Clinical Research Center of Cancer/Cancer Hospital, Chinese Academy of Medical Sciences and Peking Union Medical College, Beijing, China

**Keywords:** colorectal cancer, laparoscopy, NOSES, retrospective analysis, treatment results

## Abstract

**Introduction:**

The aim of research was to study the feasibility and safety of surgery providing specimen extraction through natural orifices in patients with colorectal cancer.

**Materials and methods:**

This study is a comparative retrospective analysis of findings obtained from 265 patients who underwent surgical treatment using NOSES technique and 275 patients who underwent laparoscopic-assisted (LA) resection. Data included preoperative patients’ information, intraoperative findings, results of postoperative pathological examination of surgical specimens, early postoperative period analysis, and follow-up.

**Results:**

Both groups were comparable in terms of gender, age and BMI. The duration of surgery was similar in both groups (*p* = 0.94). Intraoperative blood loss under NOSES interventions was slightly lower than in laparoscopic-assisted surgeries (*p* < 0.001). There was no significant difference in the number of lymph nodes removed and anal function scores between the two groups (*p* > 0.05). It was revealed that in the NOSES group, the function of the gastrointestinal tract normalized at an earlier time, slightly the time to start liquid food intake and the duration of postoperative hospital stay were reduced (*p* < 0.001). A statistically significant difference between groups was found in complications, such as pneumonia (*p* = 0.03). The absolute number of complications was observed more often in the LA surgery group (10.4%) than in the NOSES group (5.8%). Local recurrence was less common in the NOSES group (*p* = 0.01). There were no statistically significant differences in disease progression (*p* = 0.16). When analyzing disease-free and overall survival rate in this study, there was no statistically significant difference between the two surgical techniques in terms of their effect on postoperative survival (*p* > 0.05).

**Conclusion:**

The results of this study demonstrate that NOSES technique is a relatively safe and effective surgical option in patients with colorectal cancer. It has high surgical efficiency providing no increased risk of surgical intervention, reducing total number of postoperative complications, reducing duration of postoperative hospital stay, reducing the time for gastrointestinal function recovery and the start of food intake. This study supports that NOSES has clear advantages over conventional laparoscopic-assisted surgery.

## 1 Introduction

Colorectal cancer (CRC) is one of the most common malignancies, and its incidence and mortality are constantly increasing. In 2020, 555,000 new CRC cases were reported in China, accounting for 9.9% of all new malignant tumor cases and ranking the third. The number of patients among men and women was 319,000 and 236,000, respectively. Mortality from malignant neoplasms is in the fifth place, which is 12.0 per 100,000 people. CRC deaths included 165,000 men and 121,000 women, with mortality rates of 14.8 per 100,000 and 9.4 per 100,000, respectively. The incidence rate is high among the population aged 41–65 years. Over the past two decades, the incidence of colon cancer in this population has increased significantly (1). The radical treatment option for colorectal cancer is surgery, which in turn requires a constant search for new surgical approaches to ensure cardinal surgery and quality of life. Colorectal surgery has evolved over the past few decades. These changes are based on such components as minimally invasive surgical technologies, precision of intervention and organ-saving operations. Laparoscopic surgery fully meets these criteria. Currently, laparoscopic surgery has become the “gold standard” in the surgical treatment of colorectal cancer. Numerous studies globally have proven the advantage of this technique compared to open surgery. The laparoscopic technique allows reducing pain, the amount of blood loss, duration of hospitalization, and improves the cosmetic effect (2, 3). However, laparoscopic-assisted (LA) surgery requires a mini-laparotomy to extract the specimen from the abdominal cavity. The wound after mini-laparotomy is a risk factor for surgical infection and a source of pain (4). A lot of surgeons have been searching for alternative techniques to reduce surgical trauma during minimally invasive operations. The development of transluminal surgery has brought into being novel practices, new terminology has emerged. Currently, the term NOTES, in addition to completely transluminal operations, combines several types of surgical interventions: MANOS (Minilaparoscopy-assisted natural orifice surgery)—surgery through natural orifices with monoport laparoscopic assistance; LANOS (Laparoscopic-assisted natural orifice surgery)—laparoscopic-assisted surgery through natural orifices; NOSES (Natural orifice specimen extraction surgery)—implies removal of the specimen through natural openings (5–7). NOSES technique is actively developing and has gained popularity among a large number of surgeons in China, as well as in other countries. The advantage is that after the operation only a few tiny scars are left on the patient's abdominal wall, which solves the problem caused by auxiliary incisions (8, 9). This study provides relevant data from a retrospective analysis to further demonstrate the safety, feasibility, and immediate and long-term results of NOSES technique in the surgical treatment of patients with colon cancer.

## 2 Materials and methods

### 2.1 General information

We performed a retrospective analysis of the clinical findings of patients treated in the Department of Colorectal Oncology of the Second Affiliated Hospital of Harbin Medical University from 2013 to 2018. All patients met the inclusion criteria and were treated surgically; two surgical treatment options for colon cancer were compared by collecting relevant patient data. A total of 540 patients’ history cases were collected. These included 265 cases in the NOSES group and 275 cases in the laparoscopic-assisted surgery group (LA- surgery group).

### 2.2 Patient data

General patients’ data were: gender, age, body mass index (BMI), preoperative CEA and CA19-9 levels, serum albumin levels, presence of comorbidities, tumor size, neoadjuvant treatment. Surgery-related information was: operative time, intraoperative blood loss, stoma formation. Postoperative recovery and pathological data were: the start of normal functioning of the gastrointestinal tract, the time of liquid nutrition initiation, postoperative hospital stay, postoperative complications, TNM (tumor-nodes-metastasis) stage, the number of lymph nodes detected, presence of perineural, lympho-vascular and vascular invasion. Follow-up data included survival status, presence of disease recurrence or progression, and Wexner anal function score 3 months after surgery. All patients were followed up by telephone and in outpatient clinics every 3 months.

### 2.3 Inclusion and exclusion criteria

1. **Inclusion criteria:**
  a. Age >18 years;
  b. The diagnosis of colorectal cancer was confirmed by preoperative colonoscopy and pathohistological examination of biopsy material;
  c. The patient has satisfactory cardiovascular and respiratory system function. The presence of concomitant pathology does not prevent laparoscopic surgery;
  d. No history of abdominal surgery;
  e. T1 and Tis (not suitable for endoscopic resection); T2 and T3;
  f. Absence of distant metastases;
  g. Patients who gave informed voluntary consent to the personal data processing and were ready to cooperate.
2. **Exclusion criteria:**
  a. Age <18 years;
  b. Benign neoplasms of the colon and rectum;
  c. According to the results of MSCT and MRI, the presence of extensive local infiltration of the tumor and germination into neighboring organs;
  d. Primary multiple synchronous or metachronous malignant neoplasms;
  e. Presence of tumor perforation, bleeding or obstructive symptoms requiring emergency surgery;
  f. Patients who required laparotomy access conversion;
  g. Presence of severe dysfunction of the respiratory, circulatory and other systems;
  h. Incomplete information from the patient's medical history and follow-up;
  i. Lack of patient consent to the personal data processing.

### 2.4 Preoperative preparation

Prior to surgery, all patients underwent general clinical and biochemical blood tests which included detecting the level of CEA and CA19-9 tumor markers, colonoscopy with biopsy, computed tomography of the chest, abdominal cavity and pelvis, MRI of the pelvic organs to determine the nature, size, location, depth of tumor infiltration and relationship to adjacent structures and organs. For small tumors, endoscopic dye marking the tumor before surgery facilitated intraoperative visualization. The gastrointestinal tract preparation was provided 12 h before surgery. Intravenous prophylactic antibiotics was ensured 30 min before surgery.

### 2.5 Surgical technologies

Conventional laparoscopic-assisted surgery for colorectal cancer uses multicomponent balanced combined anesthesia under artificial ventilation using intravenous and gaseous anesthetics as an anesthetic support. Pneumoperitoneum is formed and pressure is maintained at 12–15 mm Hg. A 12-mm trocar for a videoscope is installed at the umbilical ring followed by examination of the abdominal cavity. After examining the abdominal organs, trocars for the working instruments are installed. Mobilization of the intestine with isolation of vascular structures is performed. This is followed by clipping and dissection of the artery and vein with lymph node dissection of the corresponding area. Further treatment of the intestinal mesentery is provided with exposure of the intestinal wall in the intended resection area. The mini-laparotomy is about 8 cm, and a section of the intestine is removed from the abdominal cavity. The intestine is resected at a sufficient distance from the tumor with assessment of the blood supply to the afferent and efferent areas. Then anastomosis is formed and patency is assessed. The abdominal wall is sutured and pneumoperitoneum is restored. Visualization of all structures is provided with testing for tightness of the anastomosis, and a drainage tube is installed.

The NOSES technique refers to laparoscopic operations, the main stages of which do not differ from laparoscopic-assisted surgeries, but the resected specimen is removed without an auxiliary incision in the anterior abdominal wall. Currently, there are three main techniques to excise and extract the specimen using NOSES technology: (1) Specimen extraction through a natural orifice (vagina or rectum) with resection outside the abdominal cavity; (2) Transanal specimen extraction by eversion with resection outside the abdominal cavity; (3) The specimen is completely resected in the abdominal cavity and removed through a natural orifice.

### 2.6 Statistical methods

In this study, the Kolmogorov-Smirnov test was used to assess the compliance of quantitative parameters with normal distribution. In cases where data did not follow a normal distribution, they were described using the median (Me) and interquartile range (Q1–Q3). Comparison of two groups for quantitative parameters that were not subject to normal distribution was carried out using the Mann-Whitney *U*-test. Absolute and percentage values were used to describe categorical variables. Analysis of percentages in four-field contingency tables was carried out using the Pearson chi-square test if expected frequencies were greater than 10, and Fisher's exact test for lower values. The quantitative measure of the effect when comparing relative values was determined through the odds ratio with a 95% confidence interval. If there were zero values in the cells of the contingency table, the calculation was adjusted using the Haldane-Anscombe correction. Comparisons of percentages in multifield contingency tables were also performed using Pearson's *χ*^2^ test. The survival function of patients was assessed using the Kaplan-Meier method. Survival analysis was performed using the Cox regression method. The optimal discriminative value of a feature was determined based on the minimum difference between sensitivity and specificity. Statistical significance of differences was determined at a significance level of *p* < 0.05.

## 3 Results

### 3.1 Comparison of the general patients’ information

We performed a retrospective analysis of surgical, combined and complex treatment of patients with colorectal cancer who received treatment in the coloproctology oncology department of the Second Affiliated Hospital of Harbin Medical University. Surgical treatment was performed by the same team of surgeons. Following the inclusion and exclusion criteria, data of 72 patients were excluded; the study included data of 540 patients. The patients’ data were divided into two groups: the group of patients who underwent surgery with the specimen extraction through natural orifices (NOSES group), which included 265 cases, and the group of patients who were exposed to laparoscopic-assisted surgery—275 cases. When comparing the general patients’ information, no statistically significant differences in gender, age, or BMI were identified between the groups (see Table 1).

**Table 1:** General patients’ information.

Parameter	Surgical option				Statistical significance of differences between groups (*p*)
	LA surgery(*n* = 275)		NOSES (*n* = 265)		
	Male patients	Female patients	Male patients	Female patients	
Gender	60.7%	39.3%	42.9%	56.8%	0.82
Age (years)	60.07 ± 1.34	61.99 ± 0.91	59.53 ± 1.17	59.54 ± 1.17	0.19
Weight (kg)	69.1 ± 2.29	59.31 ± 3.58	65.02 ± 2.34	60.06 ± 3.37	0.19
Height (cm)	171.3 ± 2.94	161.5 ± 1.82	170.6 ± 3.18	165.2 ± 2.12	0.43
Body mass index (kg/m^2^)	23.6 ± 1.02	22.8 ± 0.83	22.3 ± 2.02	22.2 ± 0.93	0.48
Diabetes	11.3%		6.4%		0.005
Hypertonic disease	22.9%		13.5%		0.0008
Cardiac ischemia	10.2%		6.4%		0.0006
Anemia	0.0%		1.5%		0.32
Hypoalbuminemia	0.4%		0.0%		0.41
Neoadjuvant chemoradiotherapy	2 (0.7%)		1 (0.4%)		0.54
Increased CEA level	62 (22.5%)		48 (18.1%)		0.2
Increased SA level 19.9	33 (12.0%)		23 (8.7%)		0.21
Descending colon	13 (4.7%)		4 (1.5%)		0.03
Cecum	2 (0.7%)		1 (0.38%)		0.49
Splenic flexure of the colon	1 (0.4%)		0 (0.0%)		0.16
Hepatic flexure of the colon	3 (1.1%)		1 (0.38%)		0.33
Ascending colon	19 (6.9%)		7 (2.64%)		0.26
Transverse colon	1 (0.4%)		0 (0.0%)		0.002
Rectosigmoid colon	1 (0.4%)		13 (4.9%)		0.08
Sigmoid colon	61 (22.2%)		49 (18.5%)		0.53
Rectum	174 (63.3%)		190(71.7%)		0.004

Analyzing the frequency of concomitant pathologies in patients (Table 1), it was revealed that in the group of laparoscopic-assisted surgery such pathologies as diabetes mellitus (11.3%, *p* = 0.0049), hypertension (22.9%, *p* = 0.0008), coronary heart disease (10.2%, *p* = 0.0006) were more common. An increased level of the CEA tumor marker occurred in 22.5% of cases (n-62) in patients of the LA surgery group, in 18.1% of cases (n-48) in patients of the NOSES group, *χ*^2^ = 0.202. Tumor marker CA 19-9 was increased in 33 patients (12%) in the LA surgery group, in 23 patients (8.7%) in the NOSES group, *χ*^2^ = 0.205. Thus, no statistically significant differences were found between the groups in the incidence of elevated levels of CEA and CA 19-9 tumor markers before surgery. No statistically significant differences between groups were revealed among patients receiving preoperative chemoradiotherapy (Table 1).

The rectum as the most common tumor location occurred in 174 cases (63.3%) in patients of the LA group, in 190 cases (71.7%) in patients of the NOSES group. Sigmoid colon cancer occurred in 61 (22.2%) patients who underwent LA surgery and in 49 (18.5%) patients who underwent NOSES surgery. The tumor located in the ascending colon occurred in 19 (6.9%) patients of LA group and in 7 (2.64%) patients of NOSES group, respectively. Other tumor localisation occurred in isolated cases (Table 1).

### 3.2 Intraoperative findings

**Figure 1:**
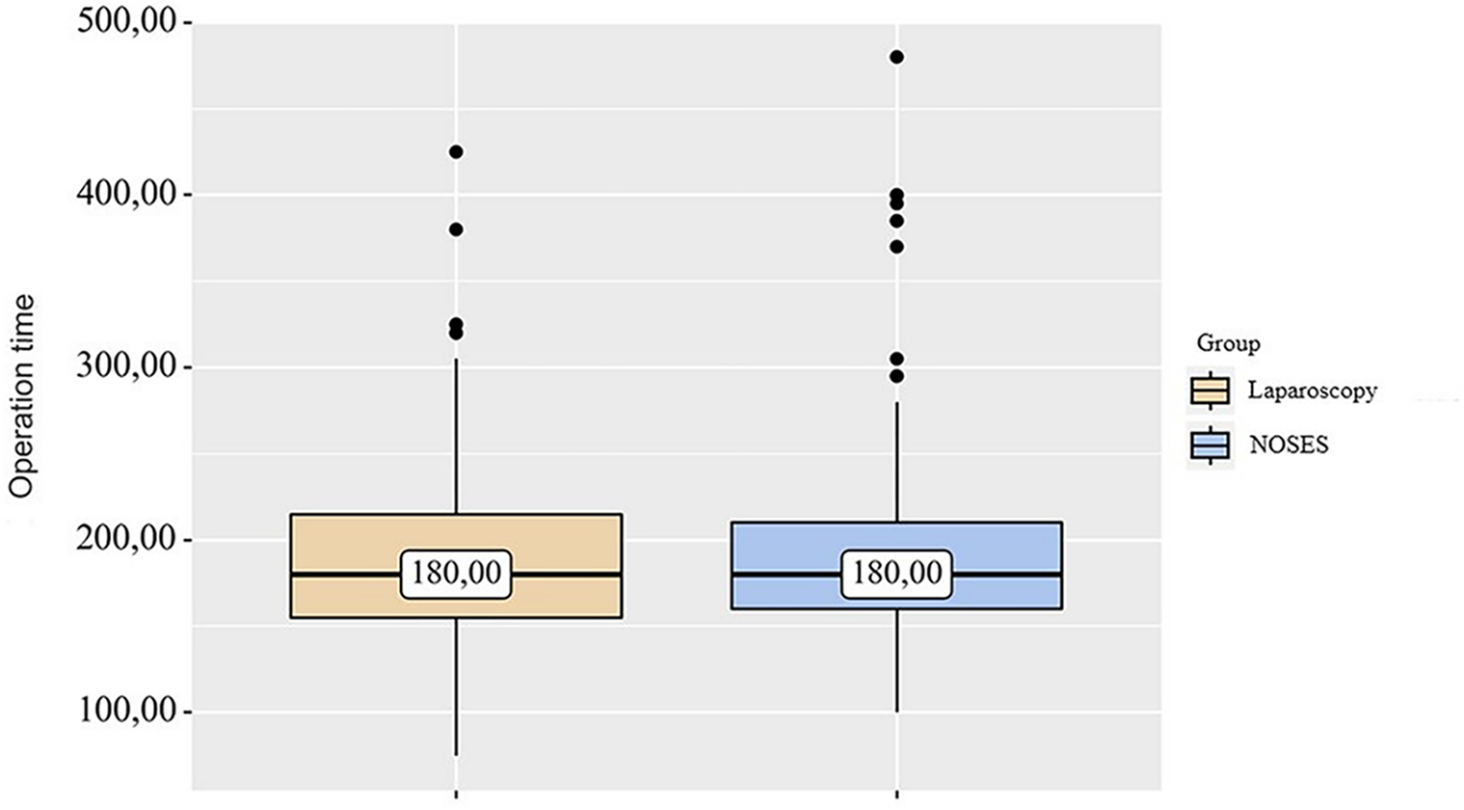
Analysis of operation time in different groups.

In the LA surgery group, 14 (5.09%) patients underwent left hemicolectomy, 25 (9.09%) patients underwent right hemicolectomy, 61 (22.18%) patients—sigmoid resection, 132 (48%) patients—anterior rectal resection, 43 (15. 64%) patients—low anterior rectal resection. In the NOSES group, right hemicolectomy was performed in 9 (3.4%) patients, left hemicolectomy was performed in 8 (3.02%) patients, and sigmoid colon resection was performed in 37 (13.96%) patients. Anterior rectal resection was performed in 100 (37.73%) patients, and low anterior rectal resection was performed in 111 (41.89%) patients. In this study, the specimen was removed through anus/vagina with resection outside the abdominal cavity in 95 (35.85%) cases. Intracorporeal resection of the specimen with extraction through anus/vagina was performed in 87 (32.83%) patients, and transanal removal of the specimen by eversion with resection outside the abdominal cavity was performed in 83 (31.32%) patients. Transanal specimen extraction was most often used—in 226 (85.28%) cases. Transvaginal extraction was used in 39 (14.72%) cases. The average duration of laparoscopic-assisted surgery was 187.73 ± 3.1 min. In the NOSES group, this parameter was 190.56 ± 3.31 min. When analyzing the operation time in different groups, no significant differences were detected *p* = 0.94 (see Table 2 and Figure 1).

**Table 2:** Analysis of operation time and intraoperative blood loss in different groups.

Parameter	Surgical options	Operation time				*p*
		Me	Q₁–Q₃		*n*	
Group	LA	180.00	155.00–215.00		275	0.94
	NOSES	180.00	160.00–210.00		265	
Parameter	Surgical option	Intraoperative blood loss				
Group	LA	50.00		20.00–100.00	275	<0.001*
	NOSES	30.00		20.00–50.00	265	

* Differences in parameters are statistically significant (*p* < 0.05).

**Figure 2:**
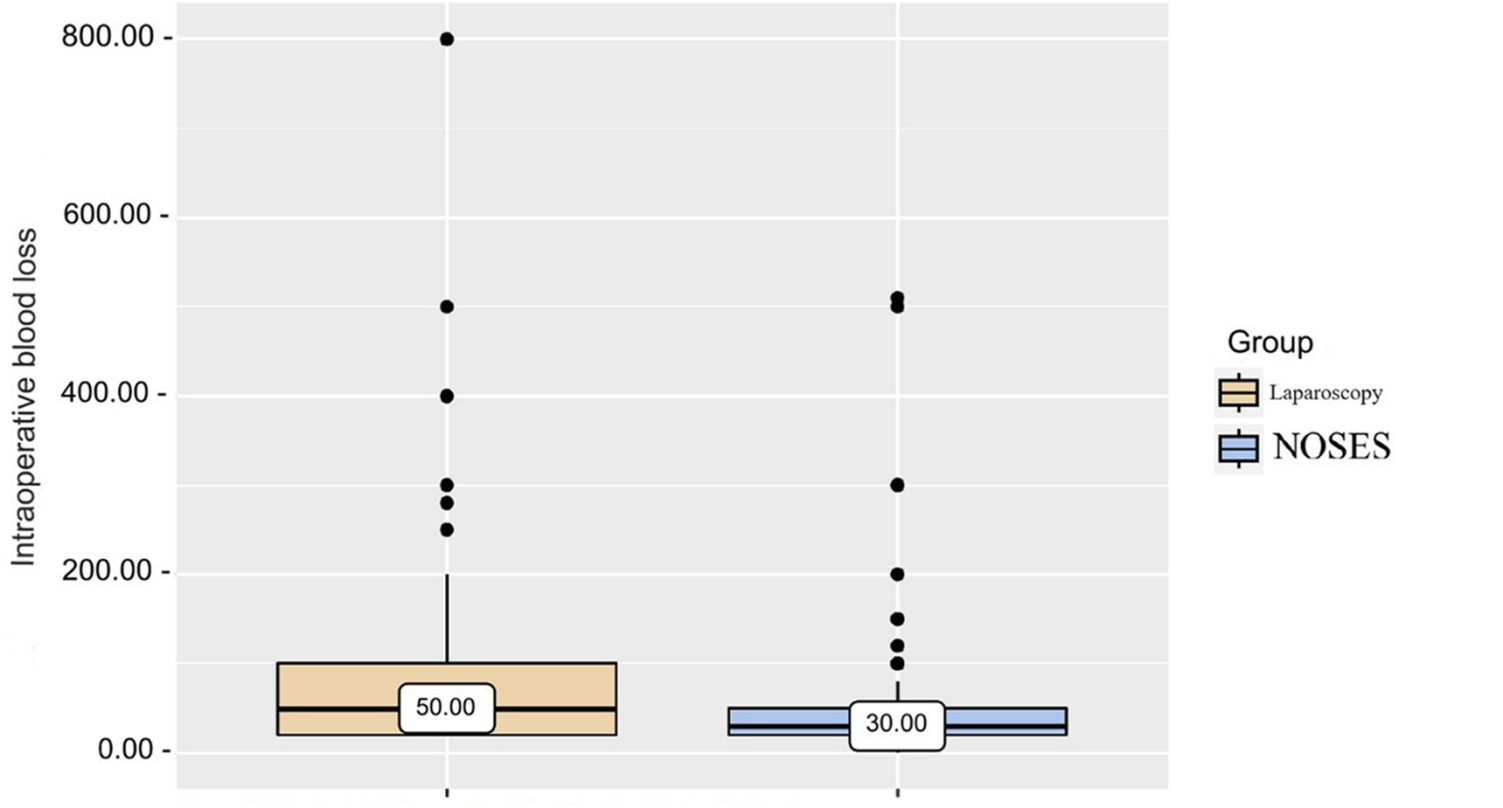
Analysis of intraoperative blood loss in patients of different groups.

Based on the data obtained from patients of different groups when analyzing intraoperative blood loss (Table 2 and Figure 2), it was revealed that in patients of the LA surgery group the volume of blood loss was slightly higher than in patients of the NOSES group, which determines a statistically significant difference in parameters (*p* < 0.001).

It was statistically significant that patients who underwent laparoscopic-assisted surgery more often needed a colostomy. In LA surgery group, stoma formation was performed in 37 cases (13.45%), and in NOSES group only in 20 (7.55%) cases (*p* < 0.05).

### 3.3 Pathomorphological data

In both groups, the tumor size was most often less than 5 cm. The predominant growth of the tumor was ulcer-like. During pathomorphological examination of surgical material, we more often observed the histological tumor type such as colorectal adenocarcinoma. There were no statistically significant differences between groups in these parameters. There were also no statistically significant differences in the number of removed and affected regional lymph nodes between the groups. In the pathomorphological material studied, the presence of vascular invasion was most often observed in patients of the NOSES group (Table 3).

**Table 3:** Pathomorphological data.

Parameters	LA	NOSES	Statistical significance of differences between groups (*p*)
Tumor size less than 5 cm	64.0%	76.7%	0.16
Tumor size 5 cm or more	36.0%	22.9%	0.23
Endophytic type	2.9%	4.5%	0.63
Exophytic type	37.8%	45.9%	0.22
Ulcer-like	56.0%	49.2%	0.76
Plaque-like	3.3%	–	–
Adenocarcinoma	94.5%	90.2%	0.63
Mucinous	5.5%	7.1%	0.22
Neuroendocrine tumor	0.0%	2.3%	0.76
Perineural invasion	57.5%	52.3%	0.24
Vascular invasion	22.2%	33.8%	0.002
Lymphatic vessel invasion	29.8%	32.3%	0.51
Total number of lymph nodes removed	14.08 ± 0.31	13.43 ± 0.34	0.23
Number of affected lymph nodes	1.03 ± 0.14	0.81 ± 0.12	0.24

There were no statistically significant differences in postoperative TNM staging for each category (Table 4).

**Table 4:** Comparasion of the process staging in the study groups.

Parameters	LA	NOSES	Statistical significance of differences between groups
Stage (St)			
0(is)	0.0%	2.6%	*χ*^2^ = 25.87; *р *= 0.001
I	26.9%	33.8%	
IIa	37.1%	22.6%	
IIb	5.1%	8.6%	
IIIa	2.9%	6.4%	
IIIb	22.2%	20.7%	
IIIc	5.8%	4.9%	
T			
Тis	0.0%	2.6%	χ^2^ = 11.23; *р *= 0.08
T1	18.5%	15.8%	
T2	12.0%	25.2%	
T3	60.7%	38.0%	
T4	8.7%	3.1%	
T4a	0.0%	13.5%	
T4b	0.0%	1.5%	
N			
N0	70.2%	68.8%	χ^2^ = 0.11; *р *= 0.95
N1	21.9%	23.9%	
N2	8%	6.7%	

### 3.4 Immediate results of treatment

Date analysis revealed that in patients of the NOSES group, the function of the gastrointestinal (GI) tract was restored at an earlier time, which allowed this group to start oral intake of liquid food slightly earlier. Postoperative hospital stay was also reduced in patients of this group (Table 5).

**Table 5:** Postoperative period.

	Postoperative time to restore GI tract functioning (hours)			*p*
	Me	Q₁–Q₃	*n*	
LA	48.00	48.00–72.00	275	<0.001
NOSES	48.00	26.00–72.00	265	
	Postoperative time to start oral intake of liquid food (hours)			
	Me	Q₁–Q₃	*n*	
LA	72.00	72.00–96.00	275	<0.001
NOSES	68.00	48.00–75.00	265	
	Postoperative hospital stay (days)			
	Me	Q₁–Q₃	n	
LA	12.00	10.00–17.00	275	<0.001
NOSES	11.00	10.00–15.00	265	

When analyzing early postoperative complications (Table 6), such as anastomotic leakage, bleeding from the anastomotic area, intra-abdominal bleeding, peritonitis, rectovaginal fistula, intestinal obstruction, deep vein thrombosis and re-operation to eliminate complications, there were no statistically significant differences in each parameter between the groups. A statistically significant difference between groups was detected in such complications as pneumonia (PA-2.2%, NOSES-0%, *p* = 0.03) and wound infection after surgery (PA-1.5%, NOSES-0%, *p* = 0, 04). However, taking into account the absolute number of complications, early postoperative complications were most often observed in patients of the LA surgery group (10.4%) than in patients of the NOSES group (5.8%).

**Table 6:** Postoperative complications.

Parameters	LA	NOSES	Statistical significance of differences between groups (*p*)
Anastomotic leakage	1.5%	3.8%	0.09
Bleeding from the anastomosis	0.7%	0.8%	0.97
Intra-abdominal bleeding	0.0%	0.0%	0.79
Peritonitis	1.1%	0.8%	0.68
Recto-vaginal fistula	0.0%	0.0%	0.66
Intestinal obstruction	1.5%	0.0%	0.05
Deep vein thrombosis	0.4%	0.0%	0.32
Pneumonia	2.2%	0.0%	0.03
Infection of a postoperative wound	1.5%	0.0%	0.05
Re-operation to eliminate complications	1.5%	0.4%	0.19

In the postoperative period, 41 patients from the LA surgery group and 38 patients from the NOSES group received adjuvant chemotherapy.

### 3.5 Long-term results of treatment and survival analysis

According to the results of a survey performed using the Wexner scale in 3 months after surgery, 17 (6.4%) patients in the NOSES group manifested postoperative anal dysfunction. In the group of patients who underwent conventional laparoscopic resection, 22 (8%) cases of anal dysfunction were noted (*p* > 0.05). Thus, no statistically significant differences were found between the groups. No vaginal dysfunction was detected in both groups.

In the long-term postoperative period, local relapse was more often observed in patients who underwent laparoscopic-assisted surgery: there were 22 (8%) cases identified; in patients of the NOSES group there were 7 (2.64%) of these cases, the fact indicating the statistical significance of the difference (*p* = 0.006, OR = 0.312; 95% CI: 0.131–0.743). Disease progression was slightly more common in patients of the NOSES group—25 (9.4%) cases. In these patients, liver metastases were the most common localisation—they occurred in 19 cases. Lung metastases were observed in 6 cases. Among patients who were exposed to laparoscopic-assisted surgery, disease progression was observed in 17 (6.2%) cases, among them, liver metastasis occurred in 13 patients, and lung metastasis in 4 patients. When comparing this phenomenon in different study groups, no statistically significant differences were revealed (*p* = 0.16, 95% CI: 0.833–3.000).

**Figure 3:**
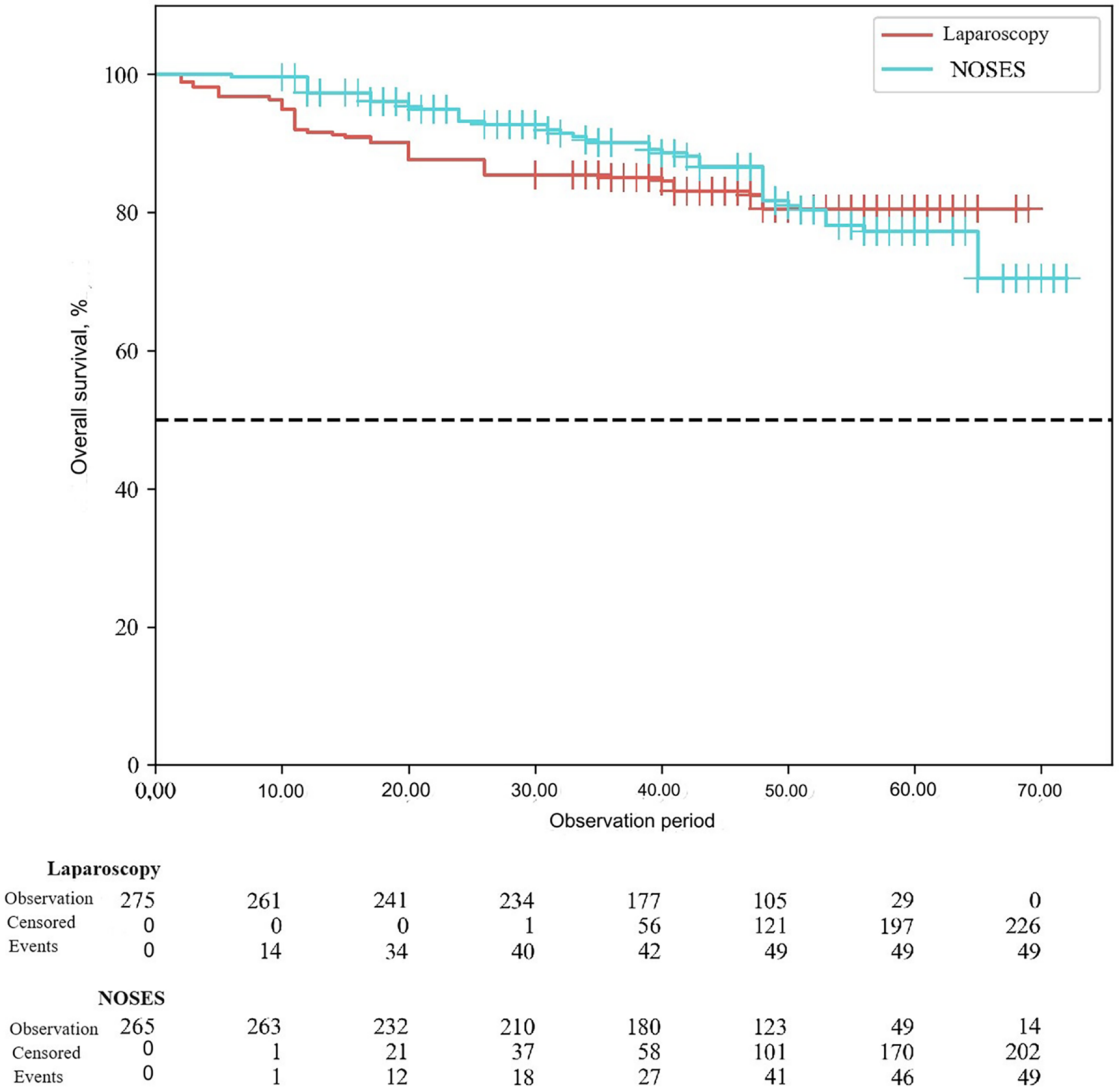
Overall survival curve in different groups.

**Figure 4:**
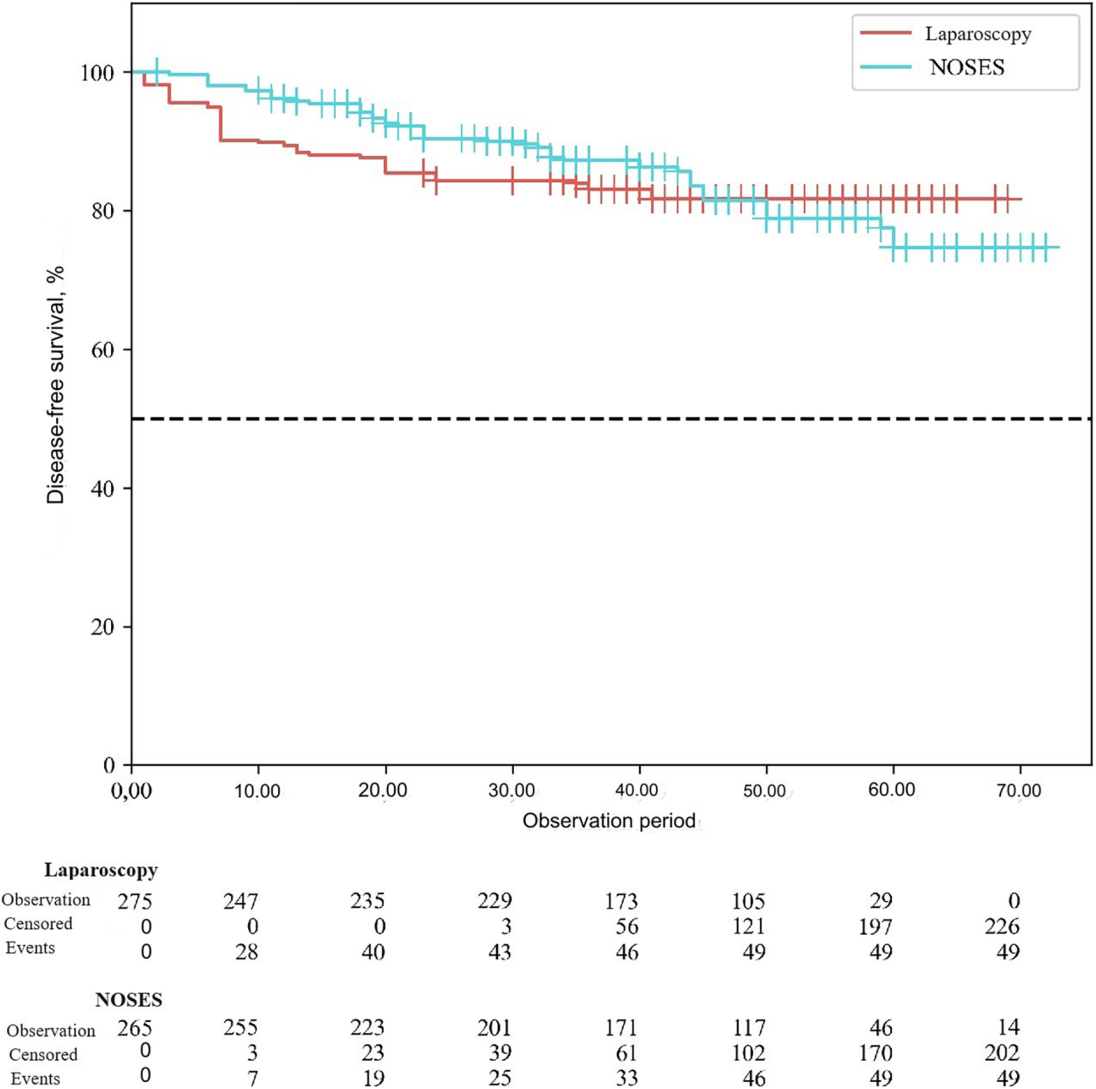
Disease-free survival curve in different groups.

According to the study results, the average duration of disease-free survival (DFS) in patients of the conventional laparoscopy group was 42.53 ± 1.04 months, and the average DFS duration in patients of the NOSES group was 44.28 ± 1.12 months (*p* = 0.25). Overall survival was 43.68 ± 0.95 months in the LA surgery group, and 45.89 ± 1.04 months in the NOSES group (*p* = 0.12). There was no significant difference in survival curves between the two groups (*p* > 0.05). Therefore, no statistically significant difference was detected between the two surgical techniques in terms of their effect on postoperative survival (See Figures 3, 4).

## 4 Discussion and conclusions

With the rapid development of minimally invasive surgical technologies, the theory of minimally invasive surgery has emerged. In 1991, Professor Jacobs (10) performed the first laparoscopic hemicolectomy, the fact representing the official introduction of laparoscopic technology into colorectal cancer surgery. Reducing the surgical incision, reducing pain in the area of the postoperative wound and reducing rehabilitation time have always been one of the goals of modern minimally invasive surgery. Laparoscopic minimally invasive surgery has become in great demand among doctors and patients and has become a routine surgical treatment for colorectal cancer. Rapid development of laparoscopic technology, improved instruments and surgeons desire to achieve excellence have resulted in the occurrence of NOSES surgery. Due to its obvious advantages, such as minimally invasive “scar-free” intervention with minimal pain, NOSES surgery has gained popularity both in China and other countries. However, the debatable issue concerning feasibility and safety of the technique still remains.

The Department of Colorectal Tumor Surgery of the Second Affiliated Hospital of Harbin Medical University is one of the first centers to perform NOSES surgery with extensive experience and favourable long-term treatment results. This study included information on patients who underwent surgical treatment in our clinic. We performed sampling based on the inclusion and exclusion criteria, and carried out a retrospective analysis of data of 540 patients. According to the literature, NOSES technique has the potential to reduce surgical trauma and pain (11–13). The development of early postoperative complications actually nullifies all the achievements of modern oncology. Despite the prevention of thromboembolic complications, the development of minimally invasive precision techniques in surgical oncology, the risk of developing postoperative complications remains high (14, 15). Most sources report a direct correlation between postoperative complications and an unfavorable immediate and remote outcome for the patient (16). Modern surgical technologies and the experience of leading surgeons do not exclude the development of postoperative intra-abdominal complications: peritonitis, intestinal suture failure, abdominal abscesses, perforations of acute ulcers, intestinal obstruction, complications from colostomy, bleeding, etc. Among the reasons for their development are an increase in the number of elderly and senile patients with severe concomitant diseases, the volume and trauma of operations performed, microbial contamination of the wound, impaired blood supply to the anastomosed ends of the intestine, errors in surgical technique (17, 18). The risk of postoperative complications in cancer patients is significantly increased due to the presence of cancer-specific factors—histogenesis and tumor size, depth of tissue invasion and the presence of metastases, as well as patient-specific factors such as toxic anemic syndrome, alimentary insufficiency (low BMI) and the state of the immune status. It has also been scientifically proven that the development of infectious and non-infectious complications is facilitated by the duration and extensive scope of surgical interventions, large intraoperative blood loss, as well as possible previous chemotherapy or radiation therapy (19). A formidable postoperative complication of colon cancer therapy is the failure of interintestinal anastomoses, causing the formation of intra-abdominal abscesses, intestinal fistulas and postoperative peritonitis and accounts for 3%–69% of the total number of postoperative complications. The authors highlight the following complications: eventration—16.7%; intra-abdominal bleeding—15.3%; necrosis of the reduced intestine—9.9%; uncontrolled widespread peritonitis—7.2%. And as a consequence of these complications, there is a high mortality rate, reaching 31% (20–27). The surgical site infection (SSI) is the most common postoperative complication after colorectal surgery, causing pain and suffering to patients. SSI remains the second most frequent type of HAI in high-income areas. It accounts for 14%–16% of hospital-acquired infections with reported rates ranged from 0.5% to 13%, depending on the type of surgery and patient characteristics (28, 29). In addition, this complication has been associated with negative economic impact, increased morbidity, extended postoperative hospital stay, readmission, sepsis, and death. Research by a number of authors indicates that following colorectal cancer procedures SSIs were significantly more common among patients over 70 years old, BMI ≥30 kg/m^2^, ASA score >2, with diabetes and chronic steroid use, undergoing open, dirty or contaminated surgery. Escherichia coli and Enterococcus spp. were the two most common pathogens isolated (30, 31). During the first postoperative day, patients should be provided with monitoring, including: (1) pulsometry, (2) ECG monitoring, (3) monitoring of the acid-base balance, coronary artery disease, and blood plasma parameters, (4) monitoring of the general biochemical blood test, blood count, and coagulogram, (5) chest x-ray, (6) monitoring of the general urine test. During the subsequent days of hospitalization, monitoring of the respiratory, cardiovascular, and digestive systems is necessary, with mandatory monitoring of the blood count and biochemical blood test. The use of inflammation markers such as C-reactive protein and procalcitonin is relevant (32). More sensitive inflammation markers are currently being sought. The authors noted the importance of presepsin concentration in the blood, which increases significantly in the presence of a bacterial infection and has a direct correlation with the stage of disease development. In addition, presepsin, acting as a predictor, can play a major role not only in determining the patient's septic state, but also in assessing the severity of the pathological process and in its prognosis (33, 34). According to a prospective study, low levels of Butyrylcholinesterase in the first and third periods after surgery were associated with an increased risk of developing SSI, which in turn shows interest in its use as a predictor of SSI development (35). The integration of new methods into the treatment of patients has important practical significance. It is worth noting the important role of the introduction of artificial intelligence in medicine. According to the literature, investigation has been conducted into the potential clinical practice implementation of deep learning algorithms for the classification and diagnosis of CRC histopathology images. Artificial intelligence and its subtypes, deep learning in particular, tend nowadays to have an expanding role in diagnosing colon cancer. Proper and early diagnosis of colon cancer is the necessary first step toward effective treatment and prevention of future disease relapse (36, 37). The absence of a laparotomy wound to remove the specimen, which is a source of pain and an entry point for infection, reduces a number of complications associated with the wound. This study demonstrates tendency of an earlier start of liquid food intake in the NOSES group is determined. Despite the fact that there was a statistically significant difference between the groups in the time of the start of the meal, the difference of 4 h was not clinically significant. There is also a statistical difference between the groups in terms of intraoperative blood loss. The average difference in intraoperative blood loss of 20 ml has no clinical significance. Operative time was not statistically different between groups. When comparing data from postoperative pathohistological examination, macro- and microscopic parameters, the number of removed and affected lymph nodes, no statistically significant difference was revealed. This fact further allows us to conclude that the quality of the resected material is similar to that obtained through laparoscopic-assisted resections.

The key factors in the development of a new surgical technique are its safety and feasibility. When assessing the safety of this promising technique, the main questions are asked: whether the principles of ablastics and antiblastics, sterility and the functional state of the anus are preserved. Stages of the operation such as opening the intestinal lumen in the abdominal cavity and inserting the head of the circular stapler into the abdominal cavity through the anus have been questioned for violating the principle of sterility. The study results demonstrated that in the NOSES group and in the conventional laparoscopic group, the cases of peritonitis that occurred were associated with anastomotic leakage. A number of essential points, such as strict bowel preparation before surgery, strict adherence to the surgical protocol, timely disinfection with iodophor gauze after cutting the intestinal tube, insertion of the head of a circular stapler into the abdominal cavity through a sterile protective sleeve, timely use of an aspirator during surgery effectively prevented the occurrence of abdominal infection cavities. There were no cases of peritoneal carcinomatosis in the NOSES group; this fact is comparable with the world literature data on the absence of significant differences in bacterial culture and exfoliative cytology of peritoneal effusion in NOSES compared with conventional laparoscopic surgery (38). A statistically significant increase in the number of complications such as postoperative pneumonia was revealed in the group of patients who underwent laparoscopic-assisted surgery (*p* < 0.05). The absolute number of postoperative complications was also significantly higher in patients of this group. It was noted that in the early postoperative period, intestinal obstruction and infection of the postoperative wound were observed only among patients who received surgical treatment using laparoscopic-assisted surgery.

The presence of anal dysfunction in patients was assessed using the Wexner scoring system in 3 months after surgery and compared between groups; and the results demonstrated that patients in both groups had no severe anal dysfunction. The incidence of postoperative anal dysfunction was insignificant (NOSES-17 (6.4%), LA-22 (8%), *p* < 0.05). This fact indicates that with careful selection of patients and strict adherence to all stages of the NOSES technique during transanal extraction of the specimen, the potential anal dysfunction can be reduced. Anal sphincter damage caused by NOSES types I, II and IV was comparable to conventional laparoscopic-assisted rectal resection.

When assessing long-term treatment results, local recurrence was statistically significantly less common in the NOSES group (*p* = 0.006). There were no statistically significant differences in disease progression (*p* = 0.16). When analyzing disease-free and overall survival in this study, there was no statistically significant difference between the two surgical options in terms of their effect on postoperative survival (*p* > 0.05).

Thus, we can conclude that with careful selection of patients and compliance with indications, the NOSES technique has the right to be the operation of choice. This technique allows avoiding a laparotomy incision, thereby reducing the level of pain, promoting early activation of the patient, earlier restoration of the gastrointestinal tract function, and reduces the duration of postoperative hospital stay. Notably, the absence of a laparotomy incision reduces the risk of wound infection and occurrence of a postoperative ventral hernia in the future. In addition, in this study, NOSES did not increase the risk of peritonitis and peritoneal carcinomatosis. There was no significant difference in the number of patients with anal dysfunction, and disease-free survival and overall survival rate after surgery were similar to results manifested by conventional laparoscopy. Based on data from the world literature and this study, we can say that the NOSES technique is safe and feasible for the radical treatment of colorectal cancer if compared to laparoscopic-assisted surgery.

## Data Availability

The raw data supporting the conclusions of this article will be made available by the authors, without undue reservation.
